# Association between exanthematous diseases and earlier age at Type 1 diabetes diagnosis: a Brazilian cohort study

**DOI:** 10.1016/j.jped.2024.11.012

**Published:** 2025-03-20

**Authors:** Lucas C.P. Lopes, Lenita Zajdenverg, Rodrigo L.M. Martins, Gabriel Araujo Medeiros, Marina D. Louro, João V.M. Lanzarin, Carlos A. Negrato

**Affiliations:** a Universidade de São Paulo, Faculdade de Medicina de Bauru, Bauru, SP, Brazil; b Universidade Federal do Rio de Janeiro, Hospital Universitário Clementino Fraga Filho, Departamento de Clínica Médica, Serviço de Nutrologia, Rio de Janeiro, RJ, Brazil

**Keywords:** Exanthematous disease, Type 1 diabetes mellitus, Cohort study

## Abstract

**Objective:**

To assess the association between exanthematous diseases, and an earlier age at Type 1 diabetes mellitus diagnosis (T1DM) in a cohort of Brazilian patients.

**Methods:**

This was a retrospective cohort study including 812 patients diagnosed with T1DM in Bauru, São Paulo, Brazil, between 1981 and 2023. Data regarding sociodemographic parameters such as age, sex, ethnicity, socioeconomic status, as well as the occurrence of a previous exanthematous diseases, such as chickenpox, measles, rubella, mumps and scarlet fever were collected. An adapted survival analysis was performed to evaluate the impact of each variable on the age of T1DM diagnosis.

**Results:**

Overall, 596 patients were evaluated. Their average age at T1DM diagnosis was 12 ± 7.69 years. It was found that presenting rubella, measles, and mumps, as well as belonging to non-high socioeconomic class, were associated with 35%, 40%, 39%, and 34% lower age at T1DM diagnosis, respectively.

**Conclusions:**

This study has found that rubella, measles, mumps, and belonging to non-high socioeconomic classes were significantly associated with earlier age at T1DM diagnosis in a cohort of Brazilian patients with T1DM. Future studies with other populations are warranted to confirm our findings.

## Introduction

Insulin deficiency is the hallmark of type 1 diabetes mellitus (T1DM). This disease derives from an interplay between genetic and environmental factors.^1^ The majority of cases, next to 90% of the total, is due to an autoimmune destruction of pancreatic β cells, while about 10% are autoantibody-negative.^1^

The incidence and prevalence of T1DM are rising worldwide, including in Brazil.^2^ Currently, nearly nine million people globally live with T1DM, and this number is expected to more than double by 2040.^2^ Additionally, around four million people are believed to have undiagnosed T1DM, with over 30,000 potentially dying within a year of disease onset.^2^^,^^3^ Over half of T1DM cases occur in individuals under 20 years old, and one-fifth are in low-income countries. In Brazil, over 100,000 people have T1DM, with projections that this number will almost double in the coming decades.^2^^,^^3^

It has been shown in many studies that patients who have T1DM diagnosed at younger ages, tend to present more diabetes-related complications and worse prognosis.^4^ Consequently, understanding the underlying pathophysiological mechanisms involved in T1DM genesis is crucial for timely diagnosis and for altering its course, improving the quality of life and life expectancy of those affected individuals.^4^

A French study used a geographical approach to map the infectious environment of children before T1DM diagnosis.^5^ It was a retrospective study that evaluated 3548 patients using data from the French Sentinel network.^5^ Associations between influenza-like infections and T1DM risk were found, while varicella infection appeared to be protective.^5^ In parallel, an Italian retrospective study evaluated the relationship between childhood infections such as measles, mumps, and rubella and T1DM from 1996 to 2001.^6^ A a significant association between T1DM incidence and mumps (P = 0.034) and rubella (P = 0.014) was found.^6^ Another Italian study, with case-control design, conducted between 1988 and 2000 found that viral childhood diseases, as measles and rubella, were directly correlated with T1DM diagnosis (OR 4.29; 95% CI, 1.57–11.74).^7^ Interestingly, an inverse correlation was observed with scarlet fever (OR 0.19; 95% CI, 0.08–0.46), though the underlying mechanism remains unclear.^7^

Therefore, the aim of this study was to assess the association between exanthematous diseases, with an early age at T1DM diagnosis in a cohort of Brazilian patients with T1DM.

## Methods

### Data source

This was a retrospective study that enrolled 812 patients diagnosed with T1DM, who received medical care at an endocrinology clinic in Bauru, São Paulo State, Brazil, from 1981 to 2023.

This endocrinology clinic attended patients from private and public systems. This private patients were those who searched for the clinic and paid for the services. The public patients were those who were referral from the Bauru's Diabetic Association, a non-profit organization focused on the reception, screening, diagnosis, and monitoring of patients with diabetes. Thus, a broad population sample was encompassed.

Included patients were those with previous T1DM diagnosis and with no age limit.

All data was provided by the patients and/or their parents and accessed through medical records evaluation.

### Data categorization

Data was categorized into two different groups. Colected sociodemographic data included age at T1DM diagnosis, sex, ethnicity, and socioeconomic status. Clinical data focused primarily on the history of exanthematous diseases of viral or bacterial etiology, such as chickenpox, measles, rubella, mumps, and scarlet fever.

T1DM diagnoses were made by physicians based in clinical protocols and guidelines issued by the Brazilian Ministry of Health, which have been periodically revised over the years. The Diagnoses encompassed classical clinical signs and symptoms, such as, polyuria, polyphagia, polydipsia, weight loss, need for insulin for clycemia controle or the occurence of diabetic ketoacidosis. Although glycated hemoglobin levels and autoantibodies may also serve as criteria for T1DM diagnoses, they were not used because they were not available to a wide range of the evaluated patients.

Ethnicity was classified as White, Black, Brown, Yellow, or Indigenous based on self-report in accordance with the classification proposed by the Instituto Brasileiro de Geografia e Estatística (IBGE).^8^ For statistical purposes, Blacks, Browns and Yellow were grouped into “Non-whites”.

Socioeconomic classification was based on the average families' monthly income. Patients were categorized into class A (greater than 20 minimum wages income), B (betweeen 10 and 20 minimum wages income), C (betweeen 4 and 10 minimum wages income), D (betweeen 2 and 4 minimum wages income) and E (less than 2 minimum wages income). his classification was based on self-report, in accordance with the criteria proposed by the Associação Brasileira de Empresas de Pesquisa.^9^ The minimum wage corresponded to the mininum wage of the year in which the patient was evaluated. For statistical purposes, classes A and B were grouped as “High-classes” and classes C to E were grouped as “Non-high classes”.

The history of previous exanthematous disease was obtained from reports made by the patients and/or their parents. They were asked about the occurence of typical symptoms, the presence/abscence of a positive serology, and positive complementary tests for each disease. We did not have access to vaccination status of the evaluated individuals.

### Data analysis

For statistical analysis, R 4.4.0 alpha Software® was used. Initially, a descriptive analysis was performed and subsequently, a modified survival analysis was carried out to evaluate the impact of each variable on the age of T1DM diagnosis (the event of interest). Hazard ratios (HR) were determined: values between 0 and 1 indicated a lower average age at T1DM diagnosis, 1 signified no change, and values above 1 indicated an increased average age at diagnosis. A p-value < 0.05 was considered statistically significant with a 95% confidence interval (CI).

### Ethical considerations

This study received approval from the Research Ethics Committee of the Bauru School of Dentistry, University of São Paulo, under protocol number: 4.872.670.

## Results

Out of 812 patients initially evaluated, only 596 patients formed the final sample. Overall, 27 individuals did not have information regarding an infection by chickenpox; 32 by measles; 26 by rubella; 45 by mumps; 18 by scarlet fever. Moreover, 29 patients chose not to declare their socioeconomic class.

The analyzed sample was formed by 310 (51.92%) women and 286 (48.08%) men; 487 (81.75%) Whites, 38 (6.35%) Blacks, 68 Browns (11.40%) 3 (0.50%) Yellows, totalizing 487 (81.75%) Whites and 109 (18.25%) non-Whites; 255 (42.71%) from high socioeconomic class, 176 (29.53%) from medium class, 120 (20.16%) from low class, and 45 (7.60%) from very low class, totalizing 255 (42.71%) from high socioeconomic class and 341 (57.29%) from non-high socioeconomic class. The characteristics of the studied sample are summarized in Table 1.

**Table 1:** Socioeconomic and demographic data of the studied patients.

Group	Evaluated patients	Average age at T1DM diagnosis	Standard deviation	Range	Hazard ratio	95% confidence interval	*p*-value
Total sample	596	12.00	7.69	1–44	–	–	–
Whites	487	12.20	7.99	1 −44	1.19	0.96–1.47	0.09
Non-Whites	109	11.10	6.13	1–29	–	–	–
Whites	487	12.20	7.99	1–44	–	–	–
Blacks	41	11.70	7.55	2–27	0.95	0.87–1.13	0.23
Browns	58	10.80	7.13	1–29	0.87	0.75–1.04	0.15
Yellows	6	11.40	7.89	2–25	0.93	0.87–1.15	0.64
Indigenous	4	10.50	7.27	1–22	0.79	0.59–1.06	0.10
Men	286	11.70	7.88	1–37	0.94	0.80–1.11	0.49
Women	310	12.30	7.49	1–44	–	–	–
High socioeconomic class	255	13.20	8.56	1–44	–	–	–
Non-high socioeconomic class	341	10.30	5.97	1–37	0.66	0.56–0.78	*p* < 0.01
A	45	12.80	7.92	1–28	–	–	–
B	210	12.70	7.56	1–44	0.98	0.85–1.11	0.21
C	150	10.20	6.32	1–37	0.65	0,42–1.06	0.16
D	130	10.10	5.80	1–33	0.62	0,57–1.04	0.19
E	61	11.30	5.15	1–29	0.73	0.59–1.02	0.08

**Figure 1:**
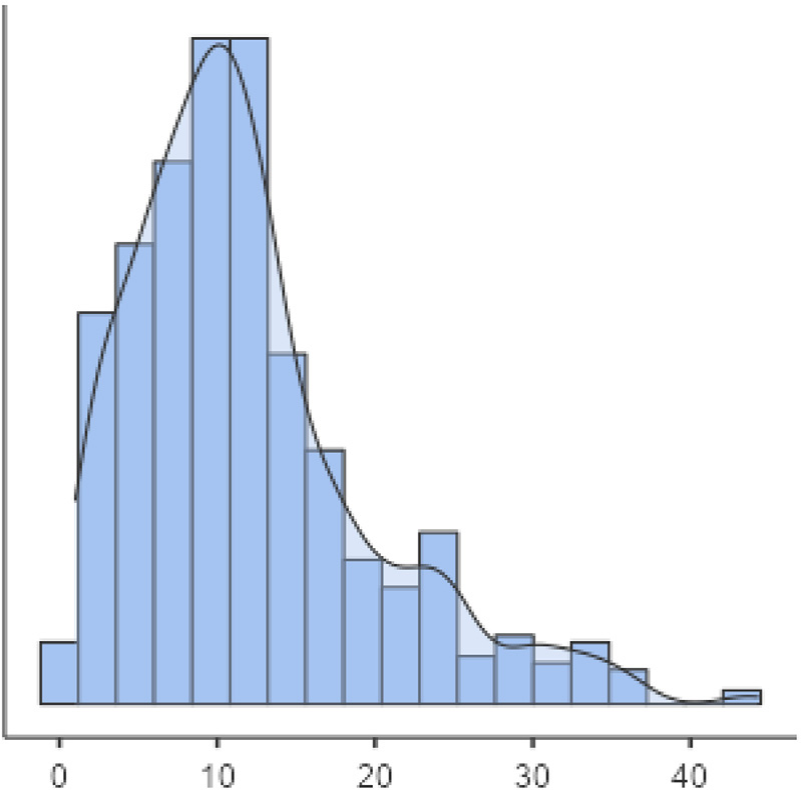
Age at type 1 diabetes mellitus diagnosis distribution. X axis, age (years); Y axis, amount.

The average age at T1DM diagnosis was 12 ± 7.69 years. Figure 1 shows the age of patients distribution. Those individuals that presented a previous diagnosis of rubeola (HR 0.65 CI, 0.51–0.84 *p* < 0.01 – Fig. 2), measles (HR: 0.60 CI, 0.48–0.74 *p* < 0.01 – Fig. 3), as well as mumps (HR: 0.61 CI, 0.50–0.73 *p* < 0.01 – Fig. 4), and those who were from non-high socioeconomic class (0.66 CI, 0.56–0.78 *p* < 0.01 – Fig. 5) tended to present a 35%, 40%, 39% and 34% lower age at T1DM diagnosis than those individuals that did not present these diseases, and were from high socioeconomic class, respectively. Figs. 2–5 can be found as supplementary material. No statistically significant differences were observed when comparing White with non-Whites. Non statistically differences were observed when comparing High socioeconomic class with non-high socioeconomic classes. The other evaluated characteristics did not show statistical significance (Table 2).

**Table 2:** Exanthematous disease infections in the studied patients.

Group	Evaluated patients	Average age at T1DM diagnosis	Standard deviation	Range	Hazard ratio	95% confidence interval of 95%	*p*-value
Previous chickenpox	154	10.90	7	1–43	0.85	0.72–1.01	*p* = 0.06
No previous chickenpox	442	15	8.72	1–44	–	–	–
Previous measles	113	11.20	7.06	1–43	0.60	0.48–0.74	*p* < 0.01
No previous measles	483	15.50	9.50	1–44	–	–	–
Previous rubella	72	11.50	7.53	1–44	0.65	0.58–0.84	*p* < 0.01
No previous rubella	524	15.60	7.99	1–34	–	–	–
Previous mumps	382	11.10	7.61	1–43	0.61	0.50–0.73	*p* < 0.01
No previous mumps	214	12.50	7.64	1–44	–	–	–
Previous scarlet fever	572	11.90	7.63	1–44	0.77	0.51–1.16	*p* = 0.21

## Discussion

### Results summary

Of 812 patients initially enrolled, only 596 were included in the final sample. It was found that, in this group, the average age at T1DM diagnosis was 12 ± 7.69 years. The evaluated variables were sex, ethnicity, socioeconomic class, and the presence or absence of a previous exanthematous disease (rubella, chickenpox, measles, mumps, and scarlet fever). Those individuals that presented rubella, measles, mumps and were from non-high socioeconomic class tended to present a 35%, 40%, 39%, and 34% lower age at T1DM diagnosis than those individuals who did not present these diseases, and were from high socioeconomic class, respectively.

### Viral factors

Our findings suggest an association between rubella, mumps, and measles infections and with an earlier age of T1DM diagnosis, as these diseases predominantly occur in childhood. An observational study conducted with Finnish children found that mumps infection could act as a trigger for the early development of T1DM which is in accordance with the results of a previously cited Italian study.^6^^,^^10^ In parallel, a review pointed that chickenpox infections could be the trigger factor for T1DM diagnoses, and not the protective factor, as mentioned by the previously cited French study.^5^^,^^11^ Higher rates of T1DM diagnoses were found in Italian regions with significant incidences of mumps, measles, and rubella. However, the impact of each one of these diaseses on T1DM diagnoses age was not discussed, unlike the other two Italian studies previously mentioned that observed a significant relationship between rubella infections and early ages of T1DM.^5^^,^^6^^,^^12^

Several hypotheses have been proposed to explain the relationship between viral infections and the age of T1DM diagnosis.^5^^,^^13^, ^14^, ^15^ It has been suggested that certain viruses may alter the expression of specific genes within the HLA class by inserting their genetic material into the host cells.^5^^,^^13^, ^14^, ^15^ Through these mechanisms, they are able to modulate genetic cell expression, promoting the synthesis of essential proteins for viral replication.^5^^,^^13^, ^14^, ^15^ Consequently, this process may inhibit the production of key human proteins, such as insulin and its receptors, ultimately contributing to the onset of T1DM.^5^^,^^13^, ^14^, ^15^

Additionally, viruses may provoke an erratic immune response, wherein antibodies mistakenly target the host's own proteins, leading to the autoimmune destruction of specific cells, such as pancreatic beta cells.^5^^,^^13^, ^14^, ^15^ This destruction results in insulin deficiency, culminating in T1DM.^5^^,^^13^, ^14^, ^15^

It has also been hypothesized that certain viruses can induce a chronic inflammatory state, which disrupts immune system modulation.^5^^,^^13^, ^14^, ^15^ This impaired immune response may prevent the effective clearance of viruses, favoring processes like apoptosis of pancreatic beta cells and fibrosis of the pancreas.^5^^,^^13^, ^14^, ^15^

Children, with their developing immune systems, are particularly susceptible to viral infections.^5^^,^^13^, ^14^, ^15^ This increased vulnerability could lead to a higher number of infected cells, alterations in protein synthesis, and a greater tendency towards chronic and erratic immune responses.^5^^,^^13^, ^14^, ^15^ Consequently, T1DM may manifest at an earlier age during childhood.^5^^,^^13^, ^14^, ^15^

Interestingly, some studies suggest that certain viral infections might confer protection against T1DM.^16^^,^^17^ This protection could be mediated by the immune response to viral infections, which involves pro-inflammatory agents such as Th1 lymphocytes and cytokines like TNF-α and interleukins 12 and 17.^16^^,^^17^ Simultaneously, immunomodulatory agents, including T helper cells, B lymphocytes, and interleukins 4 and 13, may counterbalance and regulate this immune activity, preventing uncontrolled immune attacks that lead to T1DM.^16^^,^^17^ Furthermore, upon reinfection with the same or a similar virus, the immune system responds more rapidly and effectively, preventing the onset of autoimmune mechanisms and delaying or avoiding T1DM onset.^16^^,^^17^ This may explain why we did not observe an association between a history of chickenpox and earlier T1DM diagnosis and the disagreement between the studies cited above.

Studies released in the early 2020′s decade have also reported an increase in T1DM incidence following Sars-Cov-2 infection.^18^ A systematic review and meta-analysis published in late 2022 noted that patients with a history of COVID-19 had up to a 66% higher risk of developing T1DM (Risk Ratio: 1.66, Confidence interval 95: 1.38–2.00).^18^ The spike protein of this virus may provoke a systemic inflammatory response that affects the pancreas, potentially triggering T1DM.^18^, ^19^, ^20^ A Spanish study noted that patients diagnosed with COVID-19 tended to develop T1DM at a later age possibly due to delays in recognizing T1DM symptoms or seeking medical care during the pandemic.^19^ Conversely, the SWEET Study Group observed an increase in T1DM diagnoses across various age groups but did not associate this rise with a specific age group.^20^ This may be explained by other factors, such as psychosocial stress or co-circulation of other viral agents, which also act as T1DM triggers and were influenced by the COVID-19 pandemic across different age groups.^20^

### Bacterial factors

Regarding bacterial infections, some studies suggest that certain bacteria might influence the complex interplay underlying T1DM pathogenesis.^21^ It has been proposed that bacterial agents can mimic or alter the expression of human antigens, such as HLA genes, thereby triggering or protecting against uncontrolled autoimmune responses that lead to cellular destruction and T1DM.^21^

We did not find a statistically significant association between the history of scarlet fever and early T1DM diagnosis. Interestingly, an Italian case-control study conducted between 1988 and 2000, and a Belarus retrospective cohort conducted between 1980 and 2001 reported that individuals with a history of scarlet fever tended to be diagnosed with T1DM at older ages.^8^^,^^22^ This discrepancy could be explained by individual genetic and epigenetic factors that modulate erratic immune responses to infections caused by group A beta-hemolytic Streptococcus, preventing the development of T1DM in some individuals.^8^^,^^22^

### Vaccines

Some studies suggest that immune activation induced by vaccination could potentially precipitate autoimmune reactions, acting as a catalyst for T1DM development.^23^^,^^24^ Vaccines for rubella and influenza, in particular, have been associated with an increased risk of T1DM due to their immunogenic properties.^23^^,^^24^ However, these findings remain inconclusive due to variations in sample sizes, follow-up durations, and diagnostic criteria across the studies.^23^, ^24^, ^25^

In contrast, other studies have argued that vaccines may protect against T1DM by preventing infections that would trigger excessive immune responses.^4^ Under this perspective, some studies propose that vaccines might reduce the burden on the immune system, thereby preventing the autoimmune processes that lead to T1DM.^26^^,^^27^ A Canadian case-control study developed between the 1970s and 1980s found that children who received the Bacillus Calmette-Guérin (BCG) vaccine had a lower incidence of early-onset T1DM.^26^ Additionally, a narrative review published in 2021 suggested that the idea of vaccines as T1DM triggers has been largely discussed, although ongoing discussions persist due to the incomplete understanding of certain vaccines’ immunogenic mechanisms.^27^

### Individual factors

We found an association between lower socioeconomic status and earlier age at T1DM onset. The literature, however, does not present a clear consensus linking a specific socioeconomic stratum to T1DM onset.^28^^,^^29^

A previous Brazilian study did not observe a significant difference between the socioeconomic status of the individual and the prevalence and age at T1DM diagnosis.^28^ In parallel, a cross-sectional multicenter North-American study suggested that patients from lower socioeconomic strata had worse glycemic control rates, which could impact on T1DM development.^29^

The referenced studies indicate that disparities in access to health technologies and services across different socioeconomic groups might play a significant role in the development and management of T1DM.^28^^,^^29^ Variations in access to early diagnostics, routine health screenings, and advanced care may contribute to delayed diagnosis or suboptimal disease management, potentially leading to more rapid progression of the disease in lower-income populations.^28^^,^^29^ Additionally, these disparities can influence the onset of T1DM, particularly where preventive care and timely intervention are less accessible.^28^^,^^29^

A review conducted by North-American and British researches highlighted the role of genetic risk scores across diverse ancestries in T1DM development, emphasizing that ancestry-related differences may influence the disease's onset.^30^ Similarly, a study conducted using a population-based registry from Italy discussed that sex and ethnicity may impact on T1DM development by an interaction between genes and epigenetic factors.^31^ Both studies discussed that there is no clear relationship between a specific sex and ethnicity on the risk of developing T1DM.^30^^,^^31^ This occurs because the genetic load of each group and the environmental factors that act in the modulation of these genes and may act as triggers for T1DM, vary between each location on the globe.^30^^,^^31^ Furthermore, it should be mentioned that these studies did not focus on the potential relationship between early T1DM onset and childhood exanthematous diseases, which remains an area requiring further investigation.^30^^,^^31^

### Epidemiology of the evaluated diseases

The incidence and prevalence of the diseases evaluated in this research have been decreasing over the years in Brazil.^32^^,^^33^ In fact, this derives from the Brazilian national immunization program that provides free and widely accessible vaccines to the population.^32^ Furthermore, advances in diagnosis and treatment also contribute to better control of these conditions.^33^ Unfortunately, in recent years, sporadic outbreaks of some viral diseases have been observed. In this sense, some studies conducted in Brazil observed that the prevalence of protective antibodies against certain diseases such as measles and rubella are absent in up to 20% of the target population for immunizations.^32^^,^^33^ This is possibly explained by a decrease in vaccination coverage rates in children.^32^^,^^33^

To the best of our knowledge, there is no precise epidemiological study indicating the incidence and prevalence of the exanthematous diseases evaluated in this study among the general population. However, it is suggested that cases of these conditions occur predominantly in children, with some cases in adults being associated with wild viral and bacterial strains that infect previously non-immunized individuals.^32^^,^^33^

### Limitations and strengths

One of the main limitations of this study is the potential for diagnostic and memory bias due to reliance on self-reported data, particularly in case of exanthematous diseases. Furthermore, the lack of detailed information on patients' vaccination status represented another significant limitation of this study.

This study has some stranghts that must be mentioned. All diagnoses and historical data regarding exanthematous diseases were systematically evaluated by the same medical center. Additionally, the inclusion of a wide population sample over four decades, drawn from both private and public healthcare systems, enhenced the generalizability of the findings.

The use of real-world clinical data, obtained from medical records, also enhanced the study's external validity, making the findings more applicable to general clinical practice. This study thus contributes valuable insights into the potential associations between exanthematous diseases and eralier at T1DM diagnosis, while also providing a foundation for future prospective research.

## Conclusion

This study has found that rubella, measles, mumps, and belonging to non-high socioeconomic classes were significantly associated with an earlier age at T1DM diagnosis in a cohort of Brazilian patients. Future studies with other populations are warranted to confirm our findings.

## Authors’ contributions

Lucas Casagrande Passoni Lopes participated in the data acquisition, data analysis and interpretation, and final approval of the version to be submitted; Rodrigo Lima de Meo Martins, Marina Donda Louro, Gabriel Araujo Medeiros, João Vitor Mota Lanzarin participated in data acquisition, analysis and interpretation of data and final approval of the version to be submitted; Lenita Zajdenverg and Carlos Antonio Negrato participated in study conception and design of the study, data acquistion, data analysis interpretation, drafting the article and revising it critically for important intellectual content.

## Conflicts of interest

The authors declare no conflicts of interest.

## Appendix Supplementary materials

Image, application 1

Image, application 2
