# Supplementary material for: Association between exanthematous diseases and earlier age at Type 1 diabetes diagnosis: a Brazilian cohort study
Source: J Pediatr (Rio J). 2025 Mar 20;101(3):349–55. doi: 10.1016/j.jped.2024.11.012 (PMC12039516; doi:10.1016/j.jped.2024.11.012)
Supplement: Supplementary file 1 [file mmc1.docx]

**JPED-D-24-00349_ Supplementary Material**

**Supplementary Material**

**Figure 2** Average age at type 1 diabetes diagnosis according to previous rubella diagnosis.


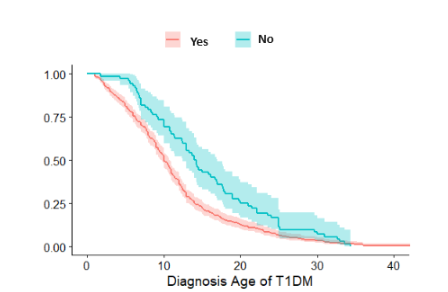

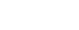


T1DM, Diabetes Mellitus Type 1. X axis represents the diagnosis age when T1DM diagnosis occurred. Y axis represents the proportion of individuals who have not yet experienced the event of interest, that is, who have not yet been diagnosed with T1DM.


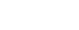

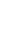


**Figure 3** Average age at type 1 diabetes diagnosis according to previous measles diagnosis.

**
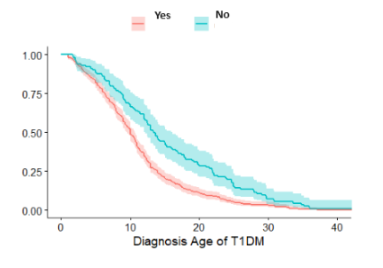
**

T1DM, Diabetes Mellitus Type 1. X axis represents the diagnosis age when T1DM diagnosis occurred. Y axis represents the proportion of individuals who have not yet experienced the event of interest, that is, who have not yet been diagnosed with T1DM.

**Figure 4** Average age at type 1 diabetes diagnosis according to previous mumps diagnosis.


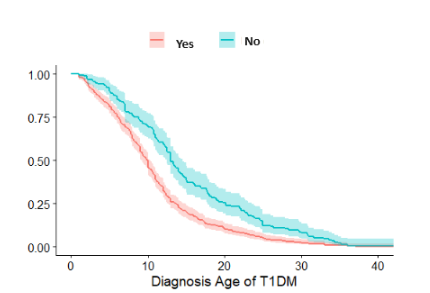


T1DM, Diabetes Mellitus Type 1. X axis represents the diagnosis age when T1DM diagnosis occurred. Y axis represents the proportion of individuals who have not yet experienced the event of interest, that is, who have not yet been diagnosed with T1DM.


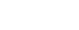


**Figure 5** Average age at type 1 diabetes diagnosis according to socioeconomic class.


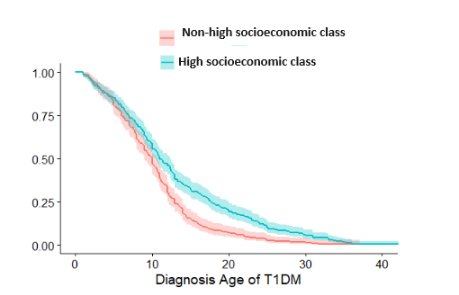


T1DM, Diabetes Mellitus Type 1. X axis represents the diagnosis age when T1DM diagnosis occurred. Y axis represents the proportion of individuals who have not yet experienced the event of interest, that is, who have not yet been diagnosed with T1DM.
