# Supplementary material for: Association between exanthematous diseases and earlier age at Type 1 diabetes diagnosis: a Brazilian cohort study
Source: J Pediatr (Rio J). 2025 Mar 20;101(3):349–55. doi: 10.1016/j.jped.2024.11.012 (PMC12039516; doi:10.1016/j.jped.2024.11.012)
Supplement: Supplementary file 2 [file mmc2.docx]

**Figure 2 - Average age at type 1 diabetes diagnosis according to previous rubella diagnosis (Supplementary material)**


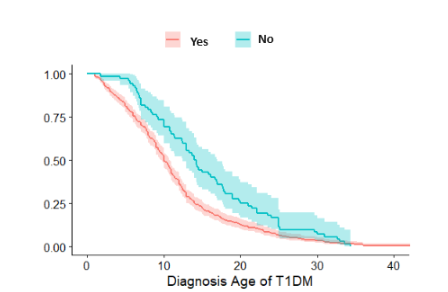

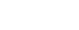


T1DM, Diabetes Mellitus Type 1. X axis represents diagnosis age when T1DM diagnosis occured. Y axis represents the proportion of individuals who have not yet experienced the event of interest, that is, who have not yet been diagnosed with T1DM.


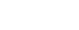

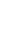


**Figure 3 - Average age at type 1 diabetes diagnosis according to previous measles diagnosis (Supplementary material)**

**
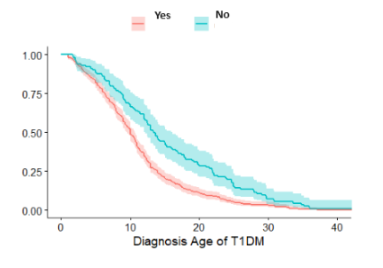
**

T1DM, Diabetes Mellitus Type 1. X axis represents diagnosis age when T1DM diagnosis occured. Y axis represents the proportion of individuals who have not yet experienced the event of interest, that is, who have not yet been diagnosed with T1DM.

**Figure 4 - Average age at type 1 diabetes diagnosis according to previous mumps diagnosis (Supplementary material)**


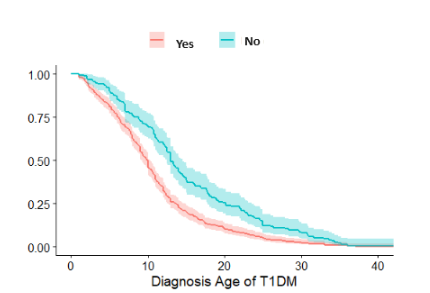


T1DM, Diabetes Mellitus Type 1. X axis represents diagnosis age when T1DM diagnosis occured. Y axis represents the proportion of individuals who have not yet experienced the event of interest, that is, who have not yet been diagnosed with T1DM.


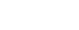


**Figure 5 - Average age at type 1 diabetes diagnosis according to socioeconomic class (Supplementary material)**


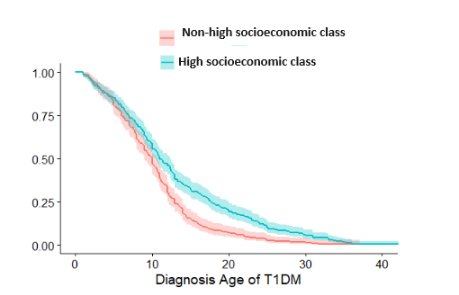


T1DM, Diabetes Mellitus Type 1. X axis represents diagnosis age when T1DM diagnosis occured. Y axis represents the proportion of individuals who have not yet experienced the event of interest, that is, who have not yet been diagnosed with T1DM.
